# Experimental dataset of the ions in mine water on mechanical strength and microstructure of Portland cement

**DOI:** 10.1016/j.dib.2025.111448

**Published:** 2025-03-07

**Authors:** Shenlong Li, Minghui Li, Guilin Yang, Fuying Shang, Junfei Zhang

**Affiliations:** a Jinjitan Mine, Shanxi Future Energy Chemical Com Ltd., Yulin 719000, China; b Envision Energy Co. Ltd., Wuxi, 214400, China; c School of Civil and Transportation Engineering, Hebei University of Technology, Tianjin 300401, China

**Keywords:** Cement, Sulfate ion, Magnesium ion, Bicarbonate ion, Compressive strength

## Abstract

The dataset presented in this paper comprises compressive strength test results for PO42.5 Portland cement mixed with mine water containing sulfate, magnesium, and bicarbonate ions. The tests are designed to systematically assess the impacts of these ions, both individually and in combination, on the long-term mechanical properties of PO42.5 Portland cement. Furthermore, X-ray diffraction (XRD) and scanning electron microscopy (SEM) analyses were conducted to investigate the microstructural characteristics of the cement-based materials under varying ionic influences. This dataset offers significant insights into the compressive strength development of cementitious materials in environments with sulfate, magnesium, and bicarbonate ion exposure.

Specifications Table

**Table:** 

Subject area	Civil Engineering
Specific subject area	Material Science, Cements and Concrete
Type of data	Table, figures
Data format	Raw, Analyzed
Data collection	Laboratory experiment (control)
Data source location	The tests was conducted at the School of Civil Engineering and Transportation Engineering, Hebei University of Technology, located in Tianjin, China.
Data accessibility	Supplementary data associated with this article can be found in the online version at https://doi.org/10.5281/zenodo.14564508 [1].

## 1 Value of the Data

• These data offer insights into the long-term mechanical behavior of concrete exposed to sulfate, magnesium, and bicarbonate ions, contributing to the development of durable concrete mixtures.
• The dataset reveals changes in the microstructure, aiding other scholars in better understanding the mechanisms behind ion-induced degradation and designing strategies to enhance the durability and elasticity of cement-based materials.
• Engineers and manufacturers can use this data to improve cement mixtures and explore sustainable cement alternatives.

## 2 Data Description

This study investigates the effects of sulfate ion concentrations (1000 mg·L⁻¹, 1500 mg·L⁻¹, 2000 mg·L⁻¹), magnesium ion concentrations (250 mg·L⁻¹, 375 mg·L⁻¹), and bicarbonate ion concentrations (600 mg·L⁻¹, 1000 mg·L⁻¹) on the strength of cement at different curing ages (7, 28, 56, 90, and 120 days). The cement was mixed with sulfate, magnesium, and bicarbonate ion solutions, and the heat evolution during cement hydration was measured using a TAM-Air isothermal calorimeter. The hydration products were characterized by X-ray diffraction (XRD), scanning electron microscopy (SEM), and thermogravimetric analysis (TG/DTG).

Table 1 presents the different experimental combinations of sulfate, magnesium, and bicarbonate ions. Table 2, Fig. 2, Fig. 3 summarizes the compressive strength of cement samples at 7, 28, 56, 90, and 120 days under various ion combinations. Figs. 1-4 illustrate the impact of different ions on the heat evolution of cement paste, while Fig. 5, Fig. 6, Fig. 7-8 show the effect of these ions on the compressive strength of the cement samples. Fig. 9, Fig. 10, Fig. 11, Fig. 12-13 display the XRD patterns of cement at 28 and 90 days for various ion conditions. Fig. 14, Fig. 15, Fig. 16, Fig. 17-18 provide SEM images of the cement hydration products under different ion influences, and Figs. 19 and 20 show the TG-DTG curves of the cement under varying ion conditions."

**Table 1:** Test mixture.

Group	Na_2_SO_4_ (SO_4_^2-^)	MgSO_4_ (Mg^2+^,SO_4_^2-^)	NaHCO_3_ (HCO_3_^-^)
Group 1	-	-	-
Group 2	1000 mg·L^-1^	-	-
Group 3	1500 mg·L^-1^		-
Group 4	2000 mg·L^-1^	-	-
Group 5	500 mg·L^-1^	Mg:250mg·L^-1^ SO_4_:1000 mg·L^-1^	-
Group 6		Mg: 375mg·L^-1^ SO_4_:1500 mg·L^-1^	
Group 7	1500 mg·L^-1^		600 mg·L^-1^
Group 8	1500 mg·L^-1^		1000 mg·L^-1^
Group 9		Mg: 375mg·L^-1^ SO_4_:1500 mg·L^-1^	1000 mg·L^-1^

**Table 2:** Compressive strength of cement under different ion combinations.

group	Compressive strength (MPa)				
	7d	28d	56d	90d	120d
1	28.3	55	59	60.8	61.4
2	38	50.3	57.1	51.4	47.6
3	40.8	45.2	50.3	45.7	40.2
4	42	43.5	48.4	41.4	39.5
5	38.4	49.3	46.7	43	41.9
6	40.3	47.2	45.1	42.5	41.3
7	34.1	51.1	49.1	46.5	44.8
8	40	53.9	47.7	45.1	43.1
9	49.1	55	47	44.5	41.3

**Fig. 1:**
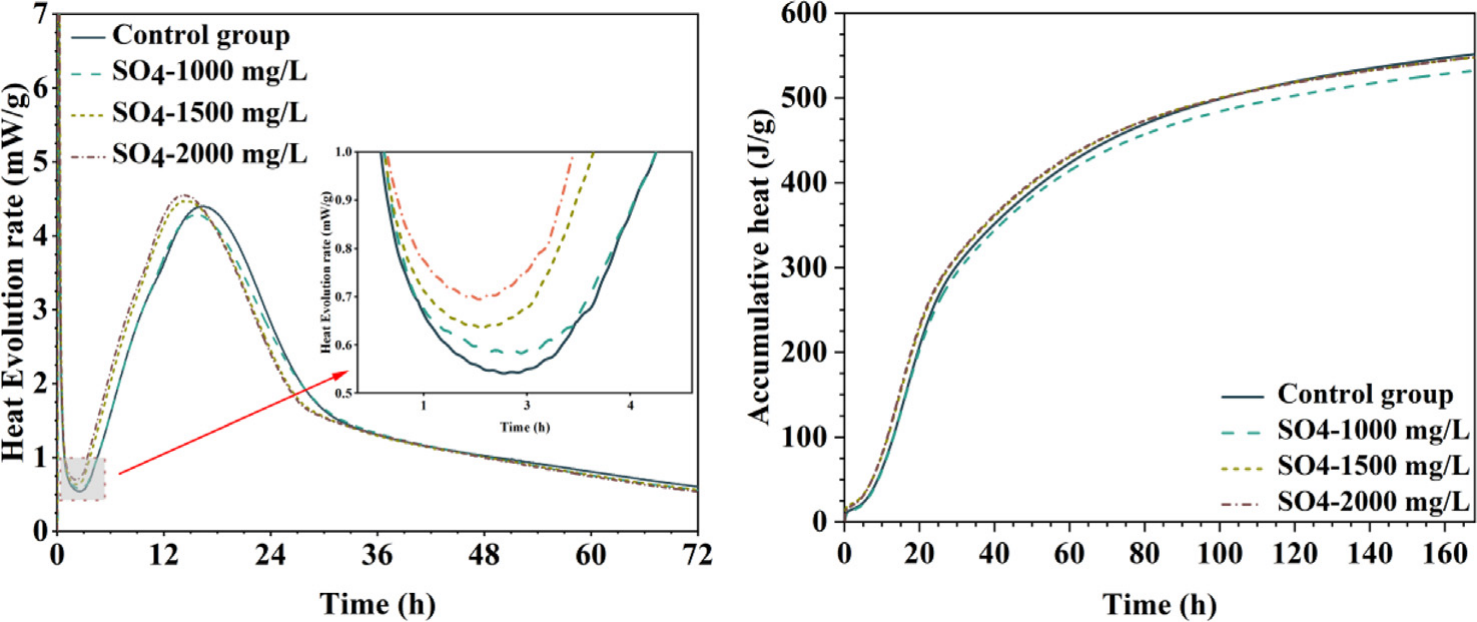
Effect of sulfate ions on the heat release rate and cumulative heat evolution of cement hydration.

**Fig. 2:**
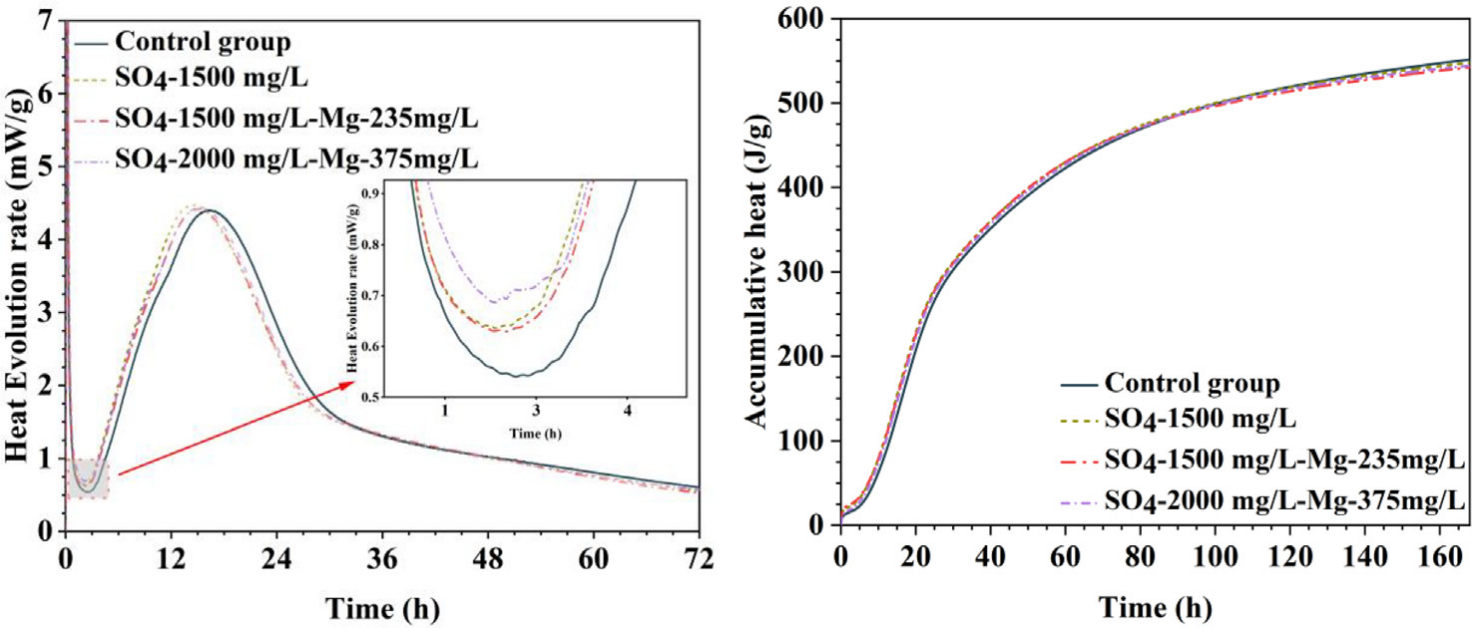
Effect of sulfate and magnesium ions on the heat release rate and cumulative heat evolution during cement hydration.

**Fig. 3:**
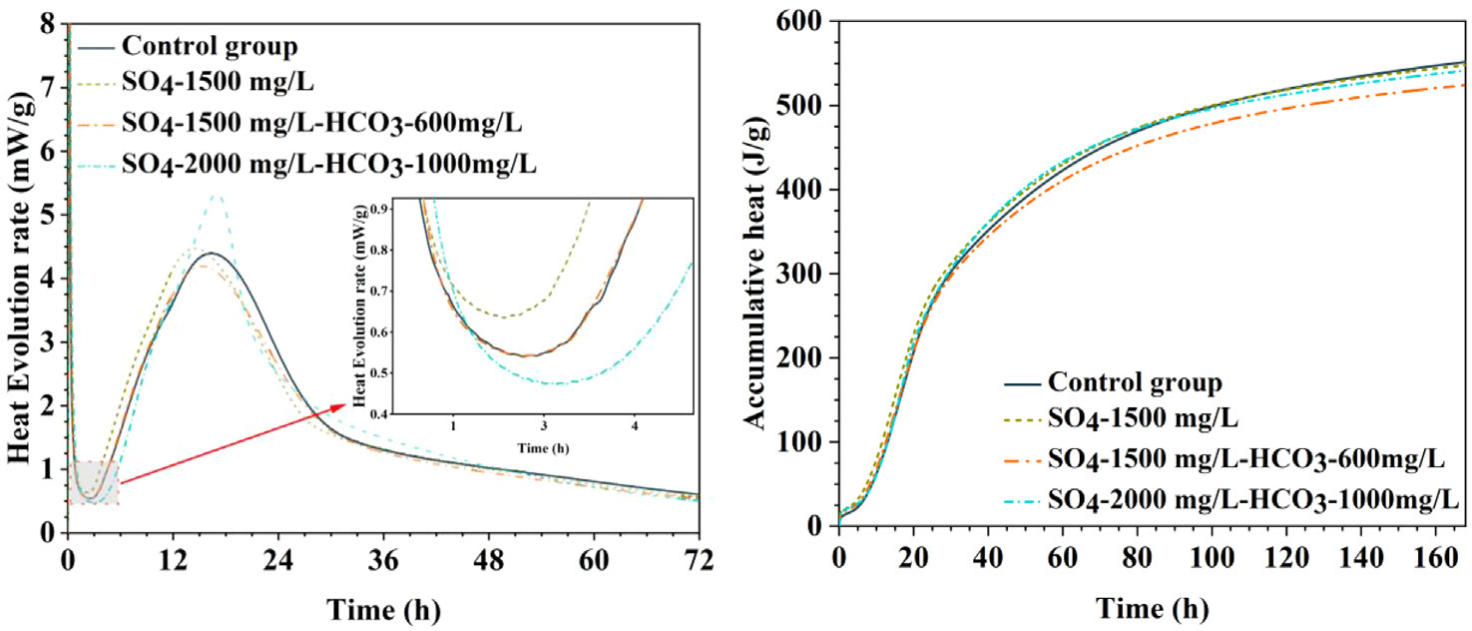
Effect of sulfate and bicarbonate ions on the heat release rate and cumulative heat evolution during cement hydration.

**Fig. 4:**
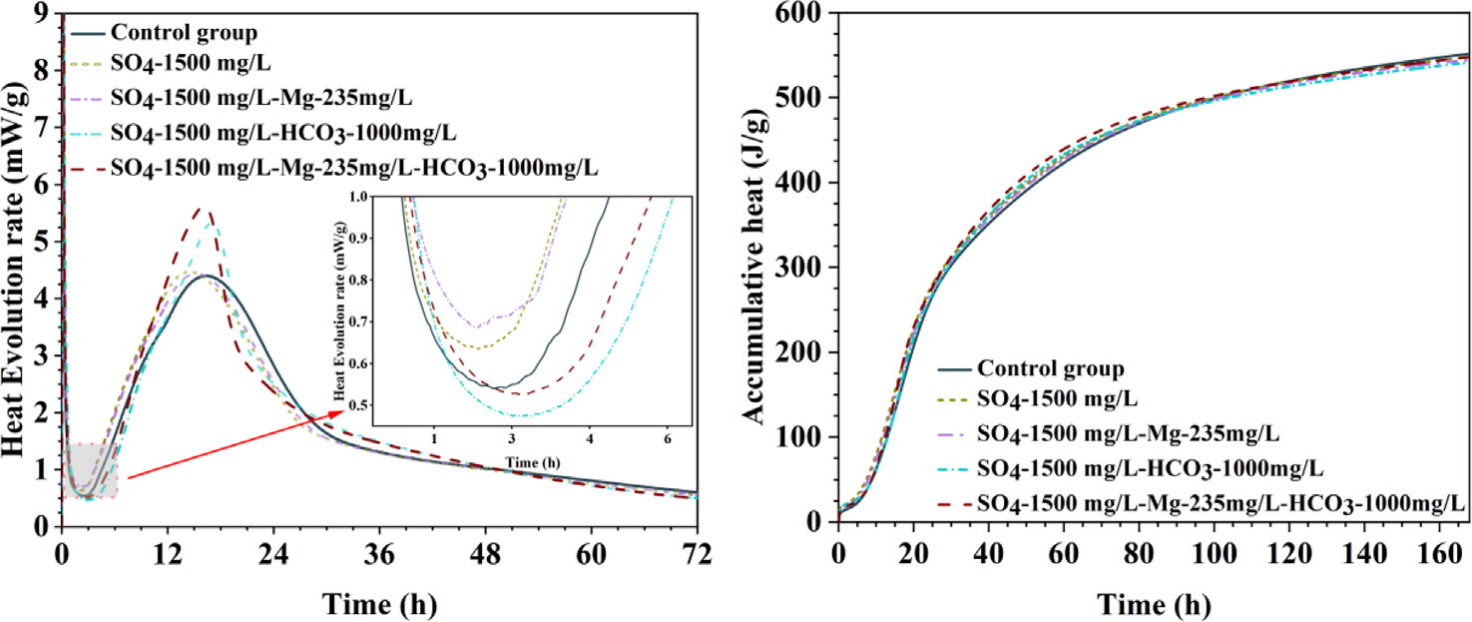
Effect of sulfate, magnesium, and bicarbonate ions on the heat release rate and cumulative heat evolution during cement hydration.

**Fig. 5:**
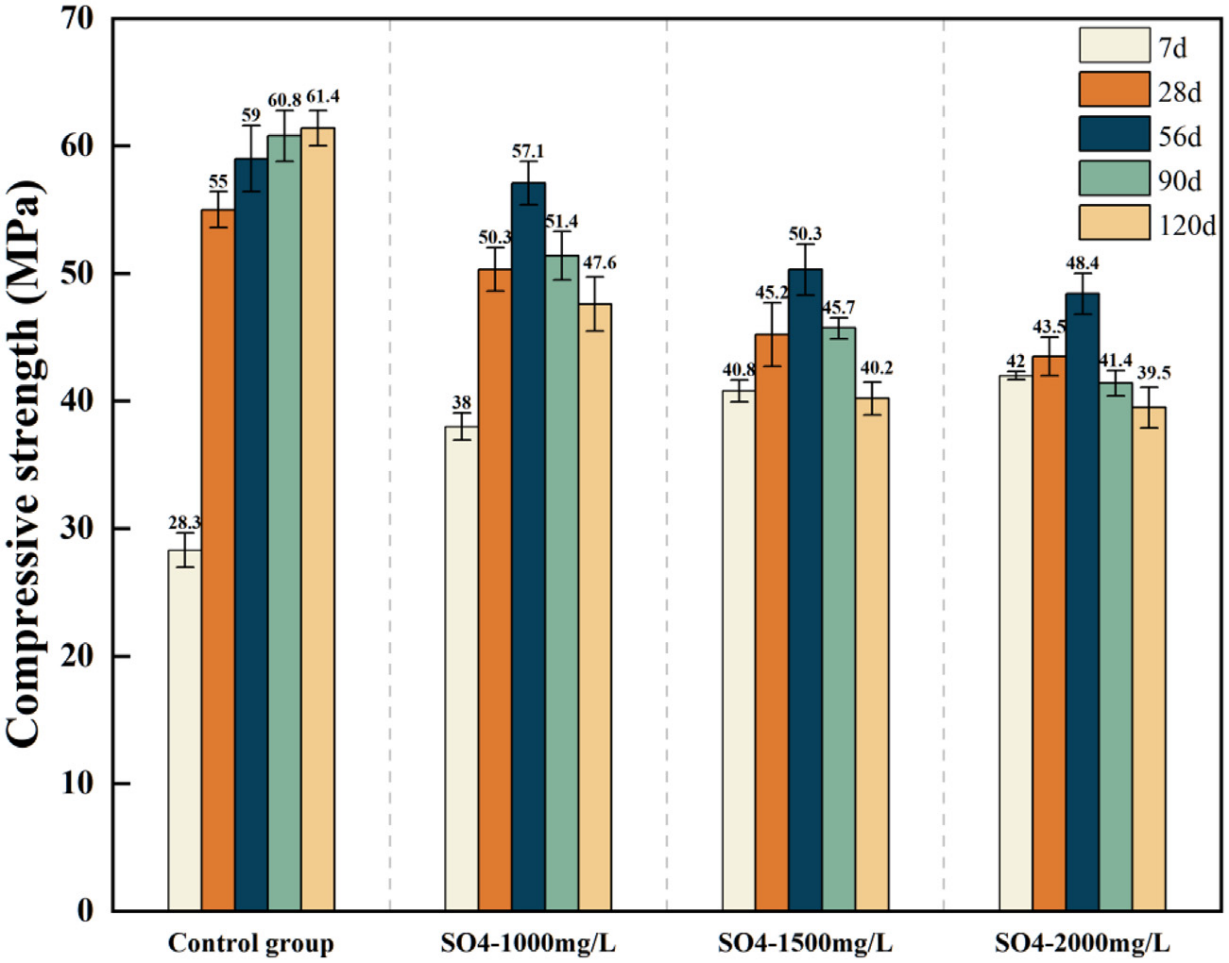
Compressive strength of cement specimens under different concentrations of sulfate ions.

**Fig. 6:**
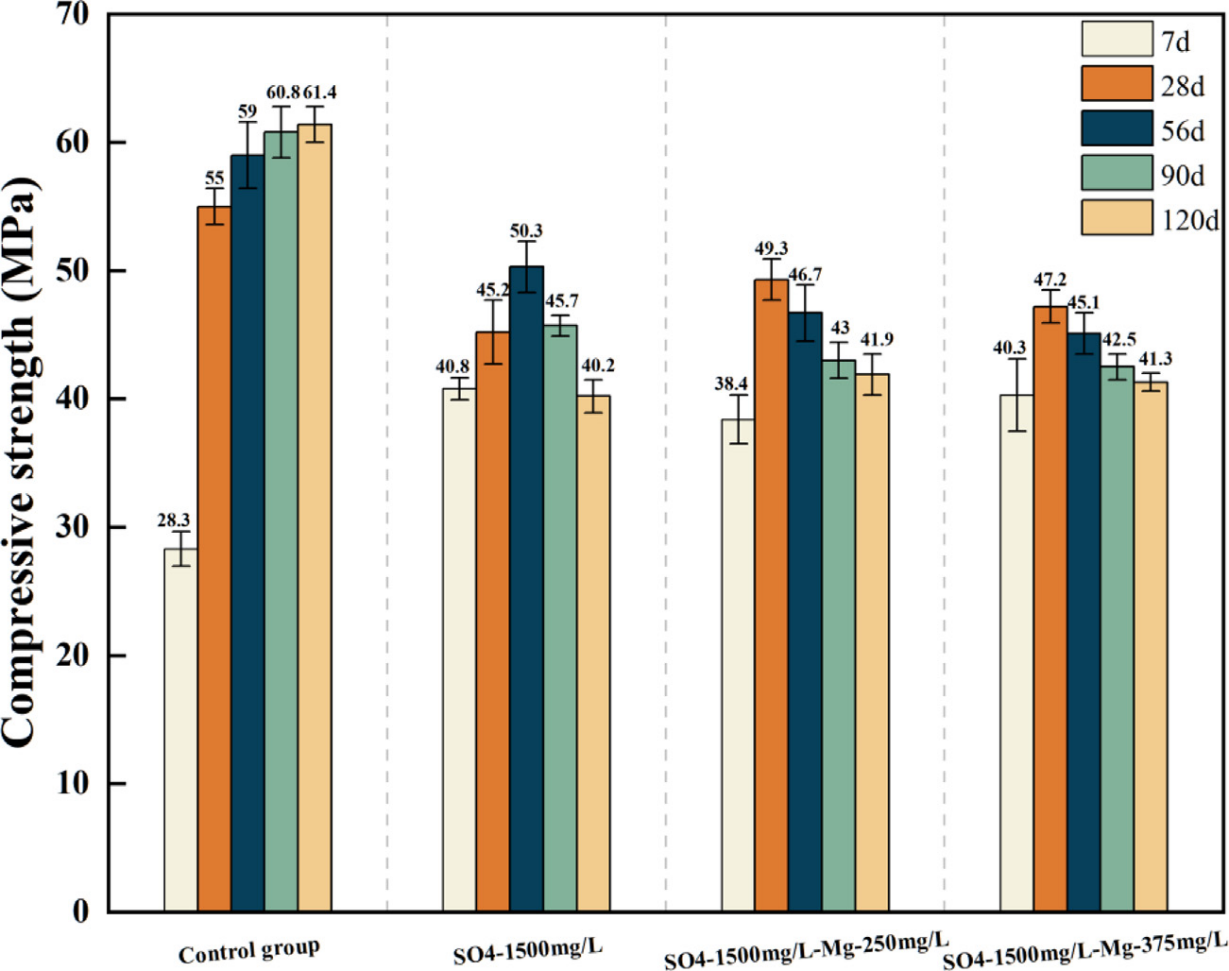
Compressive strength of cement specimens under the combined action of sulfate and magnesium ions.

**Fig. 7:**
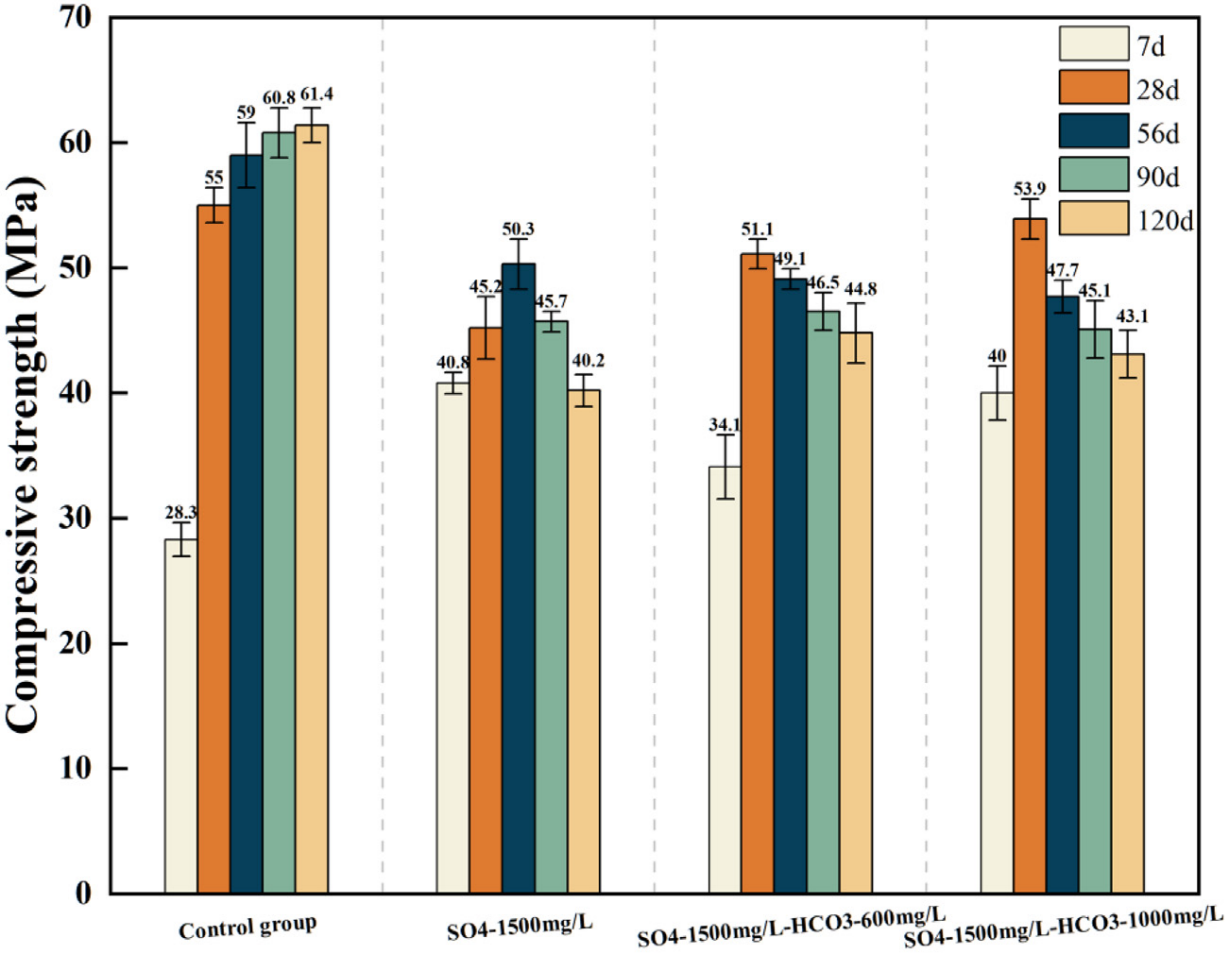
Compressive strength of cement specimens under the combined action of sulfate and bicarbonate ions.

**Fig. 8:**
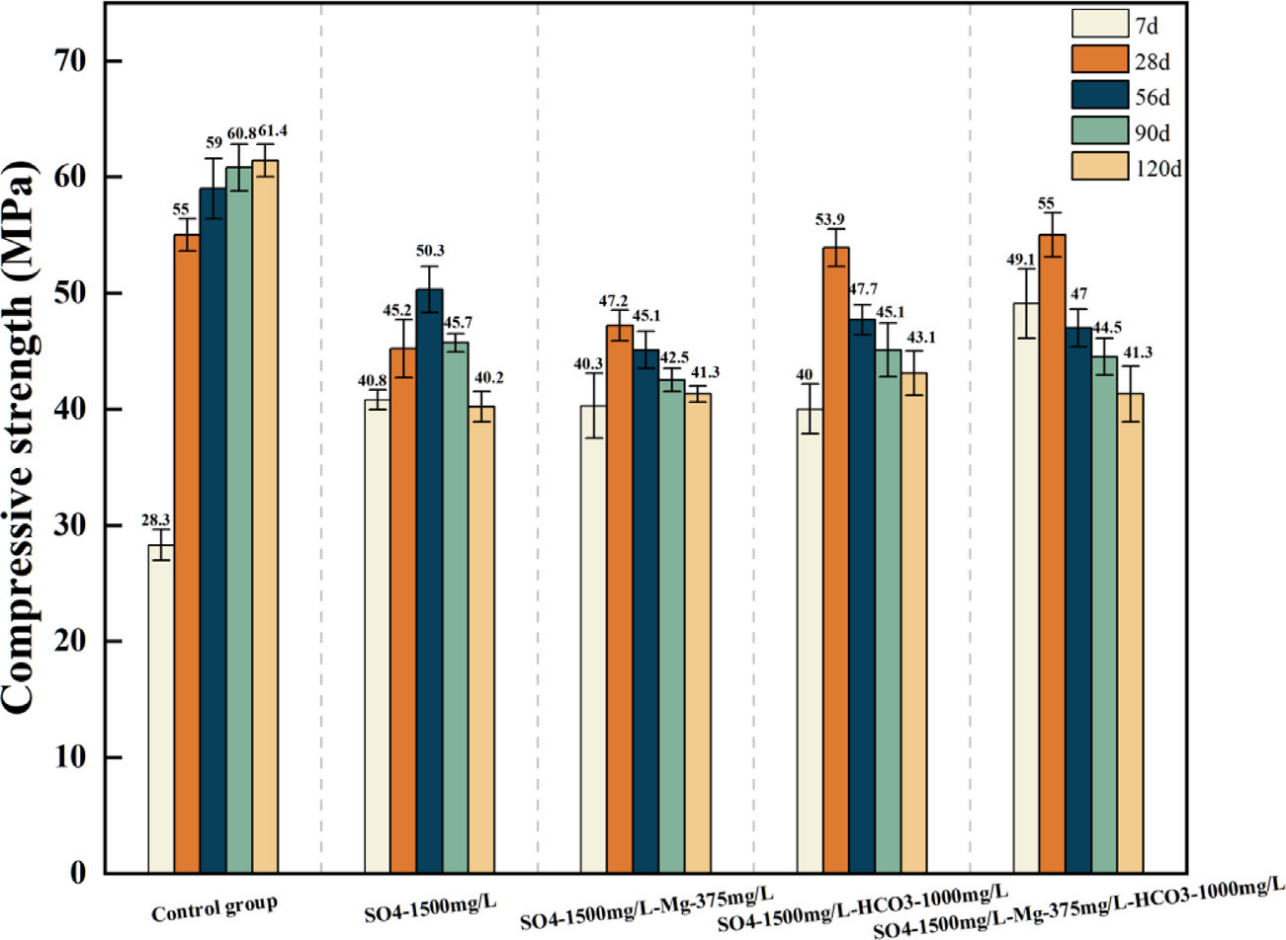
Compressive strength of cement specimens under the combined action of sulfate, magnesium, and bicarbonate ions.

**Fig. 9:**
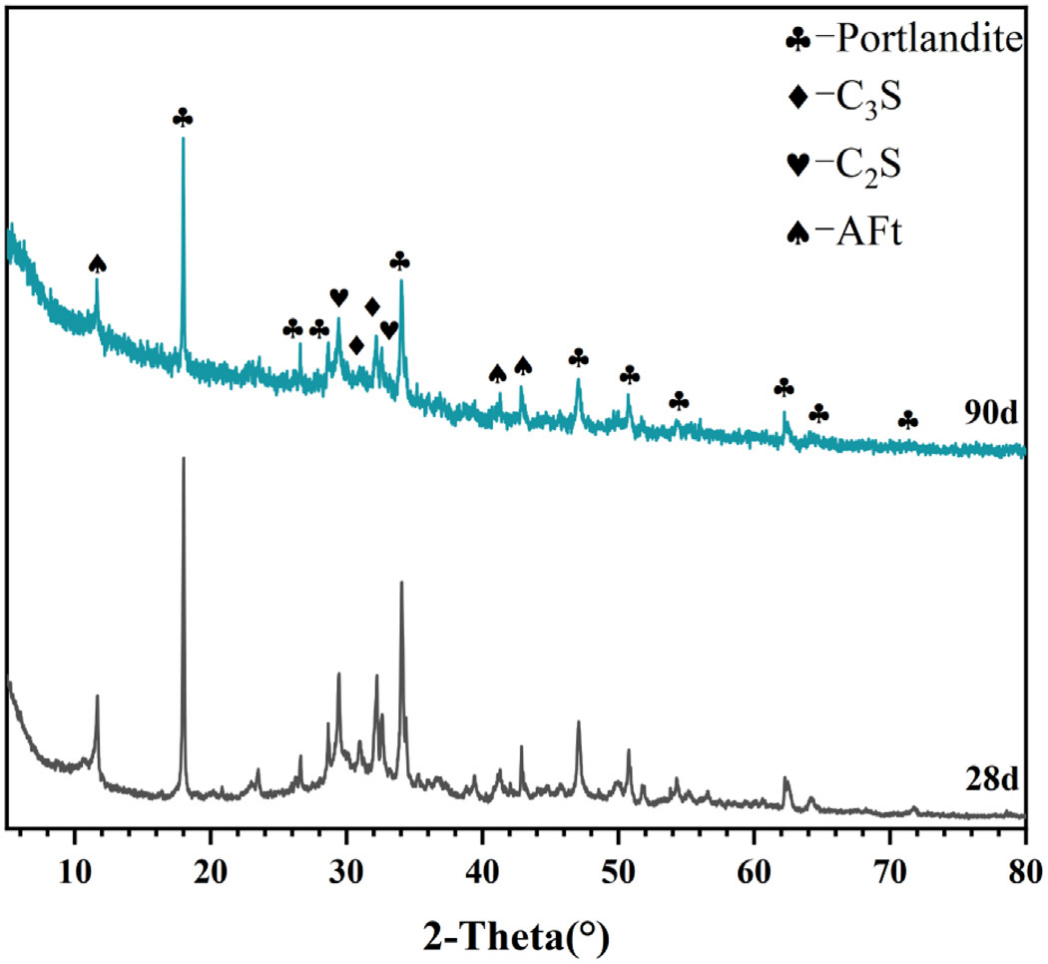
XRD patterns of the control group (Group 1) at 28 and 90 days. Identified phases and corresponding PDF card numbers: Portlandite (PDF# 04-0733), C₃S (PDF# 49-0442), C₂S (PDF# 33-0302), and AFt (PDF# 41-1451).

**Fig. 10:**
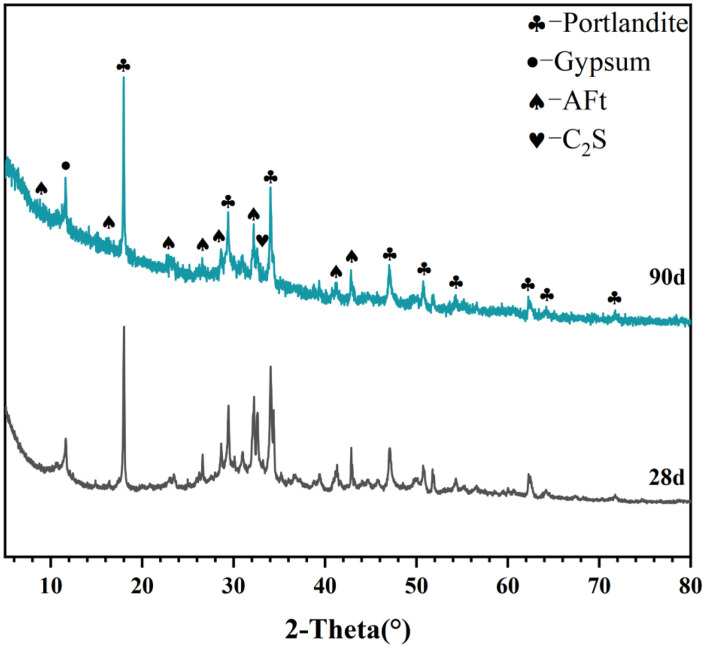
XRD patterns of cement under the influence of sulfate ions (Group 3) at 28 and 90 days. Identified crystalline phases and corresponding PDF card numbers are: Portlandite (PDF# 04-0733), Gypsum (PDF# 33-0311), AFt (PDF# 41-1451), and C₂S (PDF# 33-0302).

**Fig. 11:**
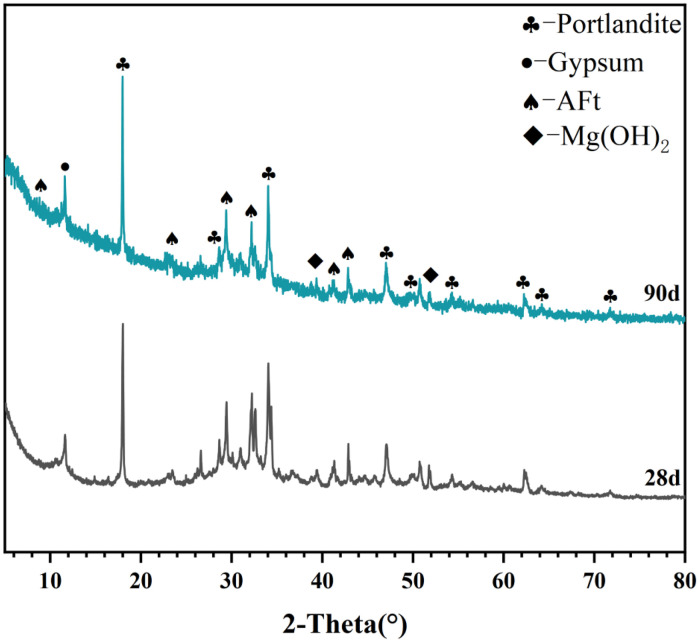
XRD patterns of cement under the influence of sulfate and magnesium ions (Group 6) at 28 and 90 days. Identified crystalline phases and their corresponding PDF card numbers are as follows: Portlandite (PDF# 04-0733), Gypsum (PDF# 33-0311), AFt (PDF# 41-1451), and Mg(OH)₂ (PDF# 07-0239).

**Fig. 12:**
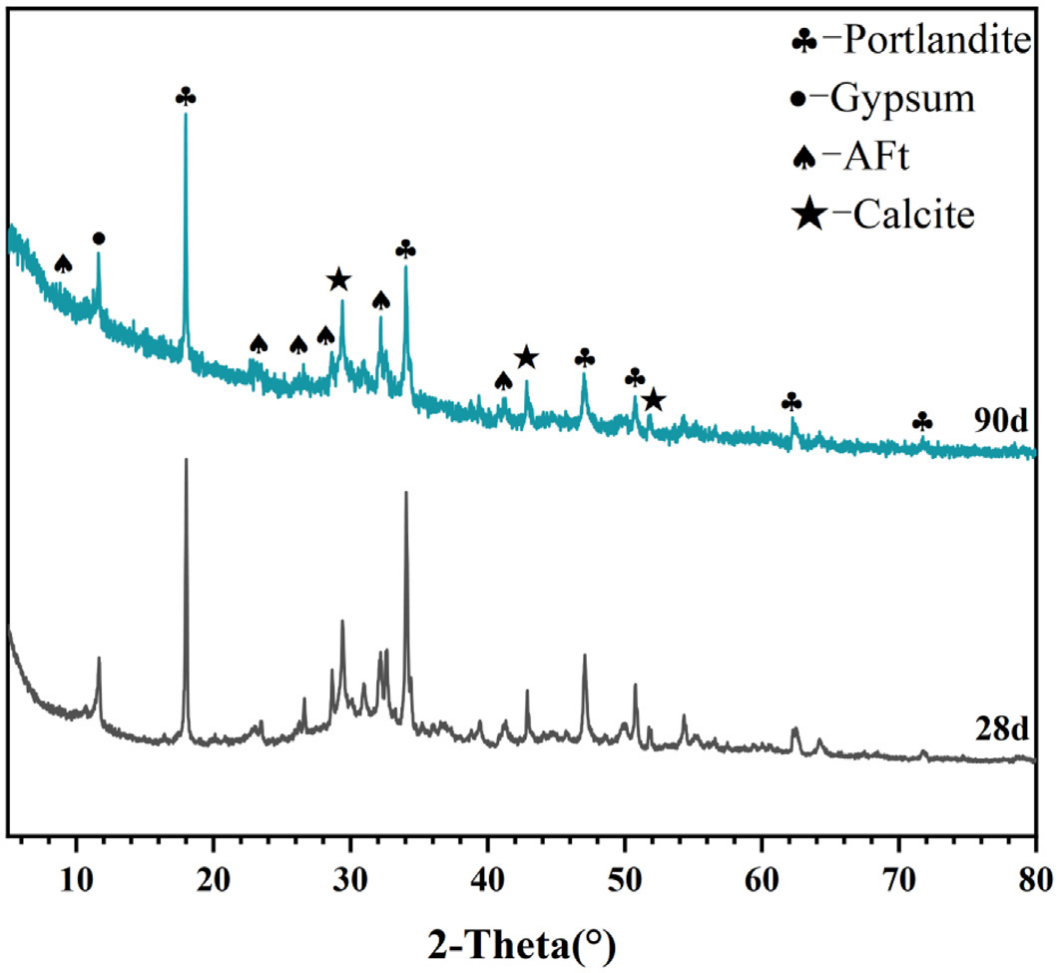
XRD patterns of cement under the influence of sulfate and bicarbonate ions (Group 8) at 28 and 90 days. Identified crystalline phases and corresponding PDF card numbers are as follows: Portlandite (PDF# 04-0733), Gypsum (PDF# 33-0311), AFt (PDF# 41-1451), and Calcite (PDF# 05-0586).

**Fig. 13:**
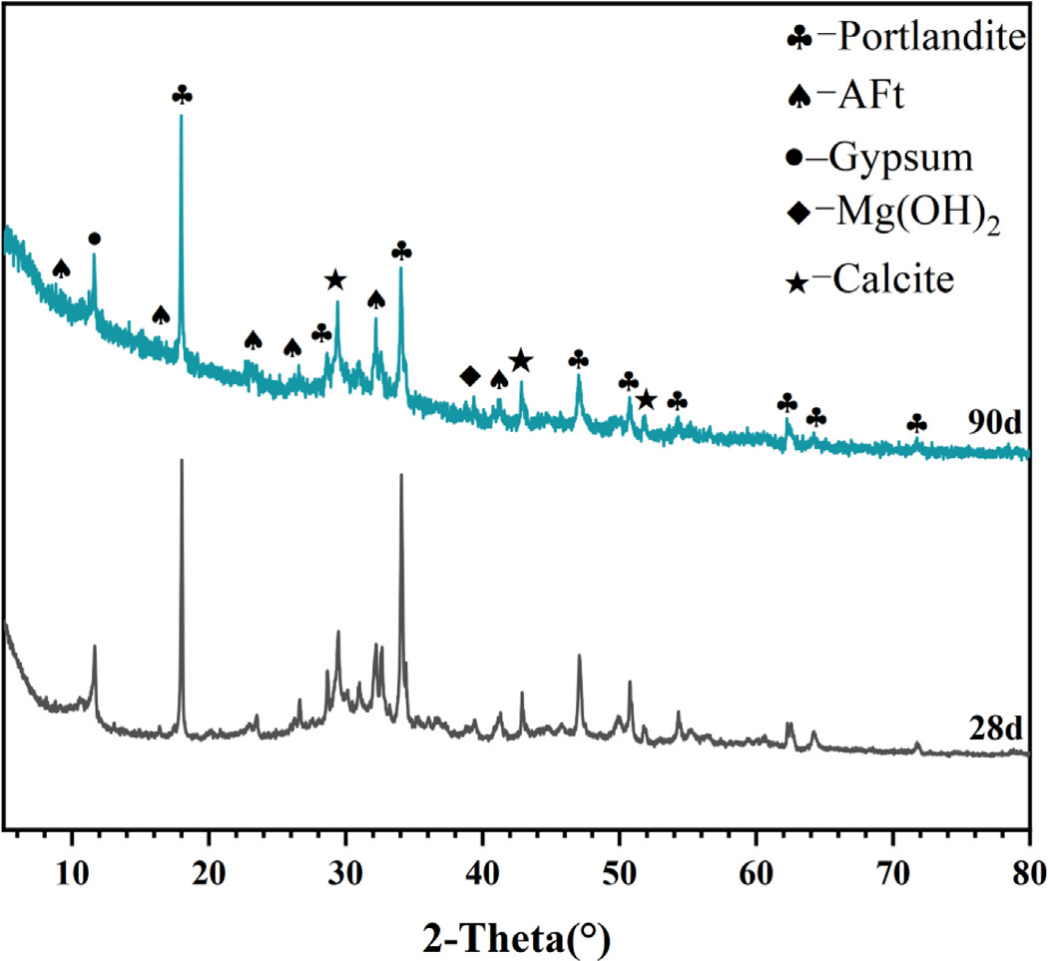
XRD patterns of cement under the influence of sulfate, magnesium, and bicarbonate ions (Group 9) at 28 and 90 days. Identified crystalline phases and their corresponding PDF card numbers are as follows: Portlandite (PDF# 04-0733), AFt (PDF# 41-1451), Gypsum (PDF# 33-0311), Mg(OH)₂ (PDF# 07-0239), and Calcite (PDF# 05-0586).

**Fig. 14:**
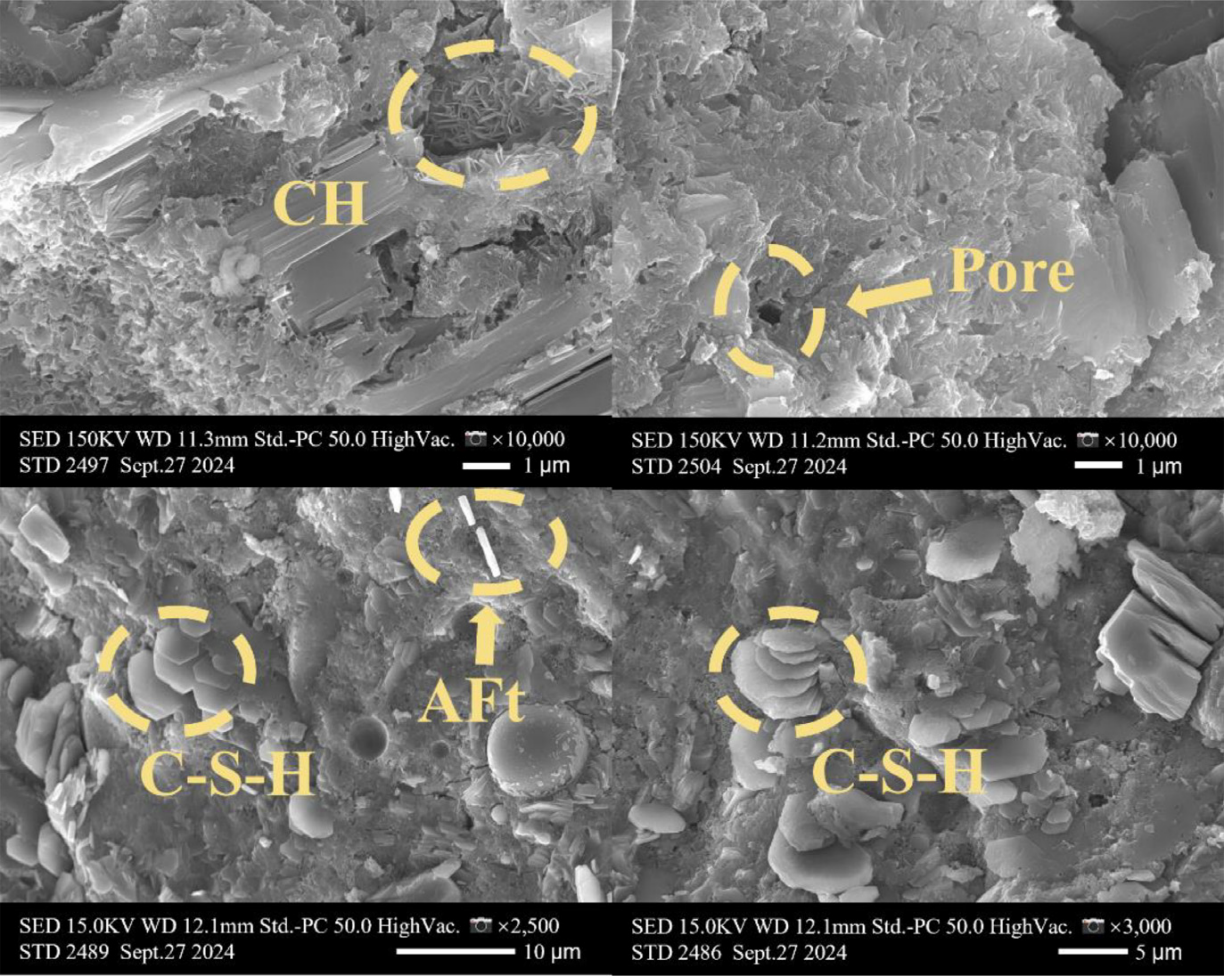
SEM images of hydration products of the control group (Group 1) at 28 days.

**Fig. 15:**
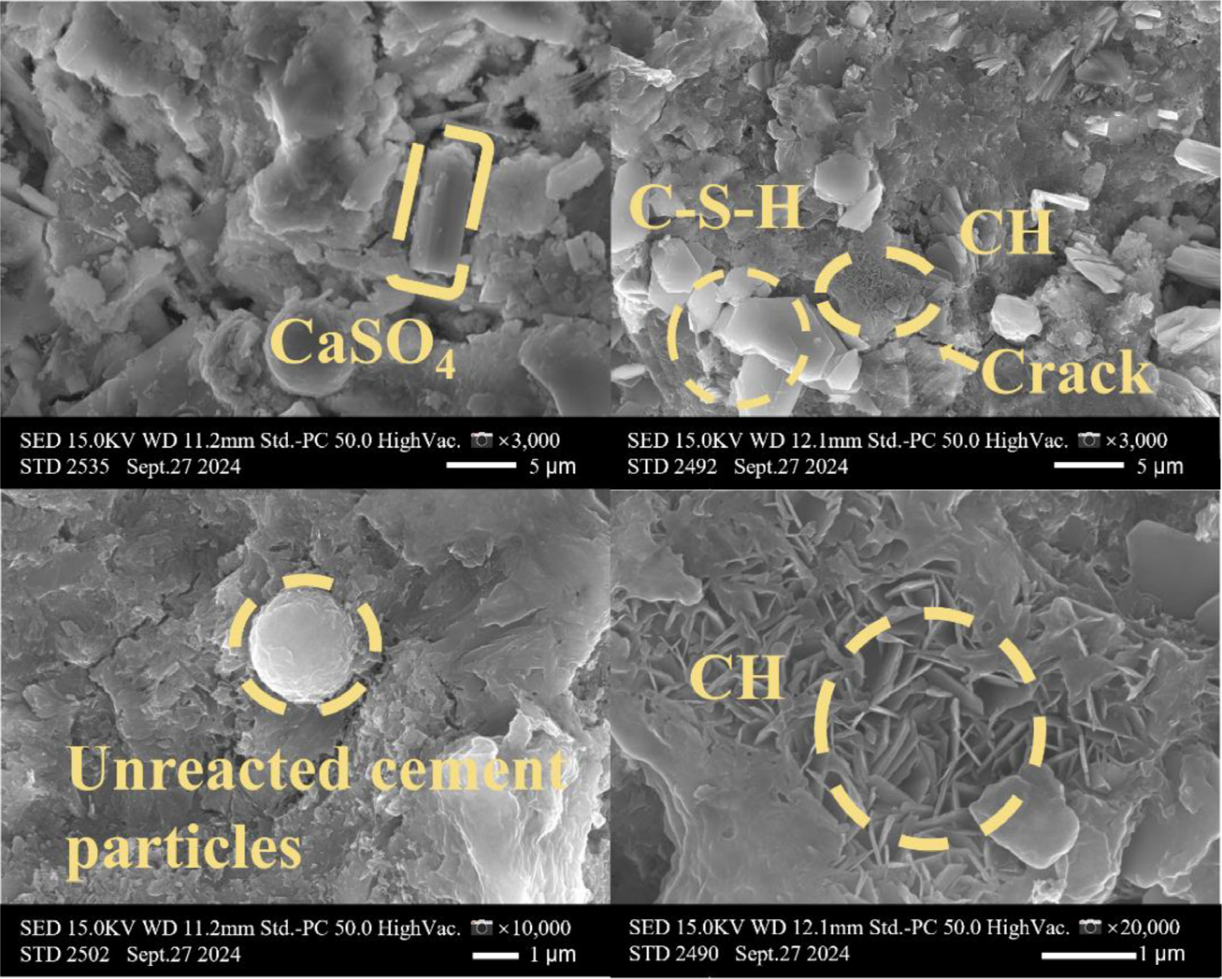
SEM images of hydration products of cement under the influence of sulfate ions (Group 3) at 28 days.

**Fig. 16:**
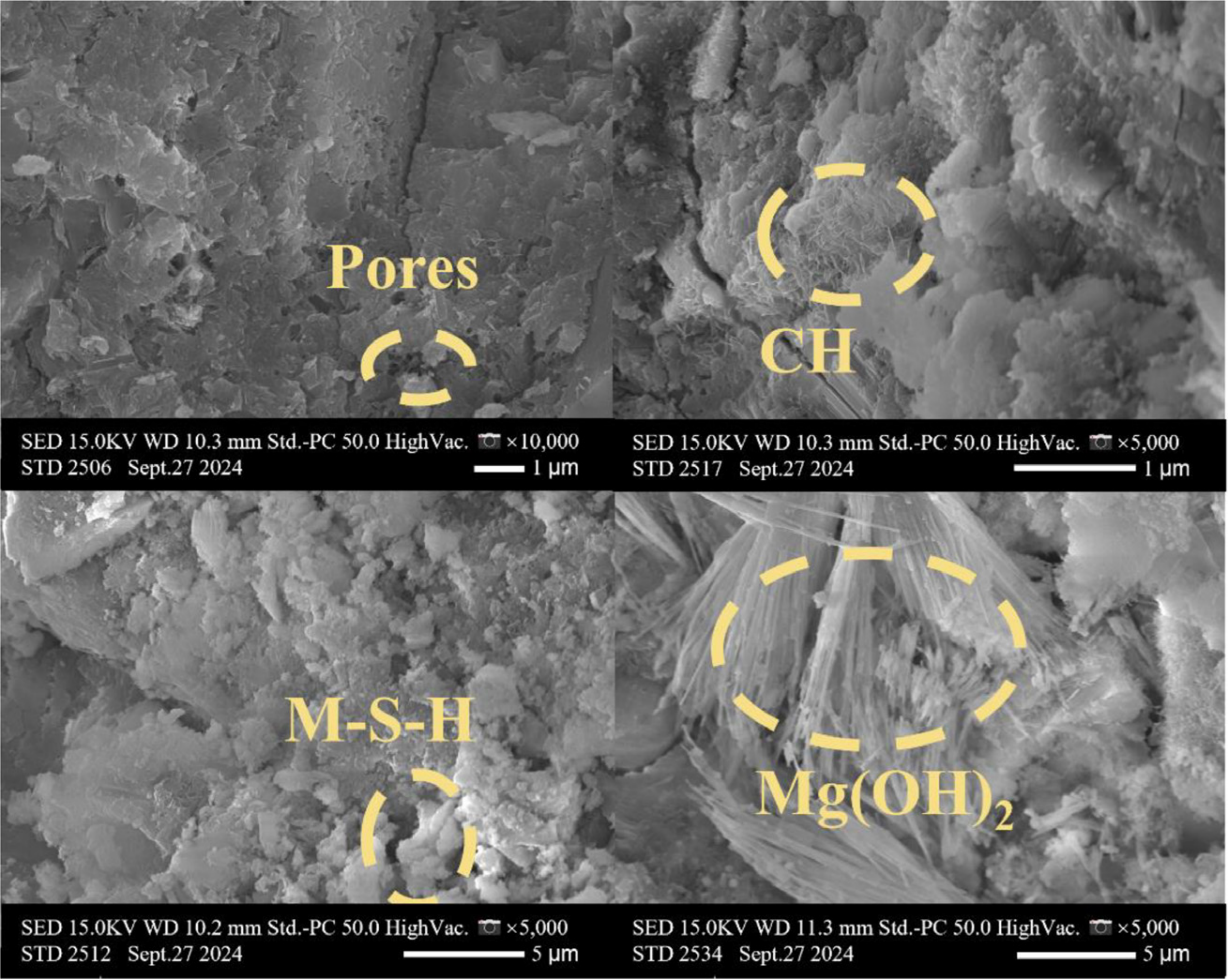
SEM images of hydration products of cement under the influence of sulfate and magnesium ions (Group 6) at 28 days.

**Fig. 17:**
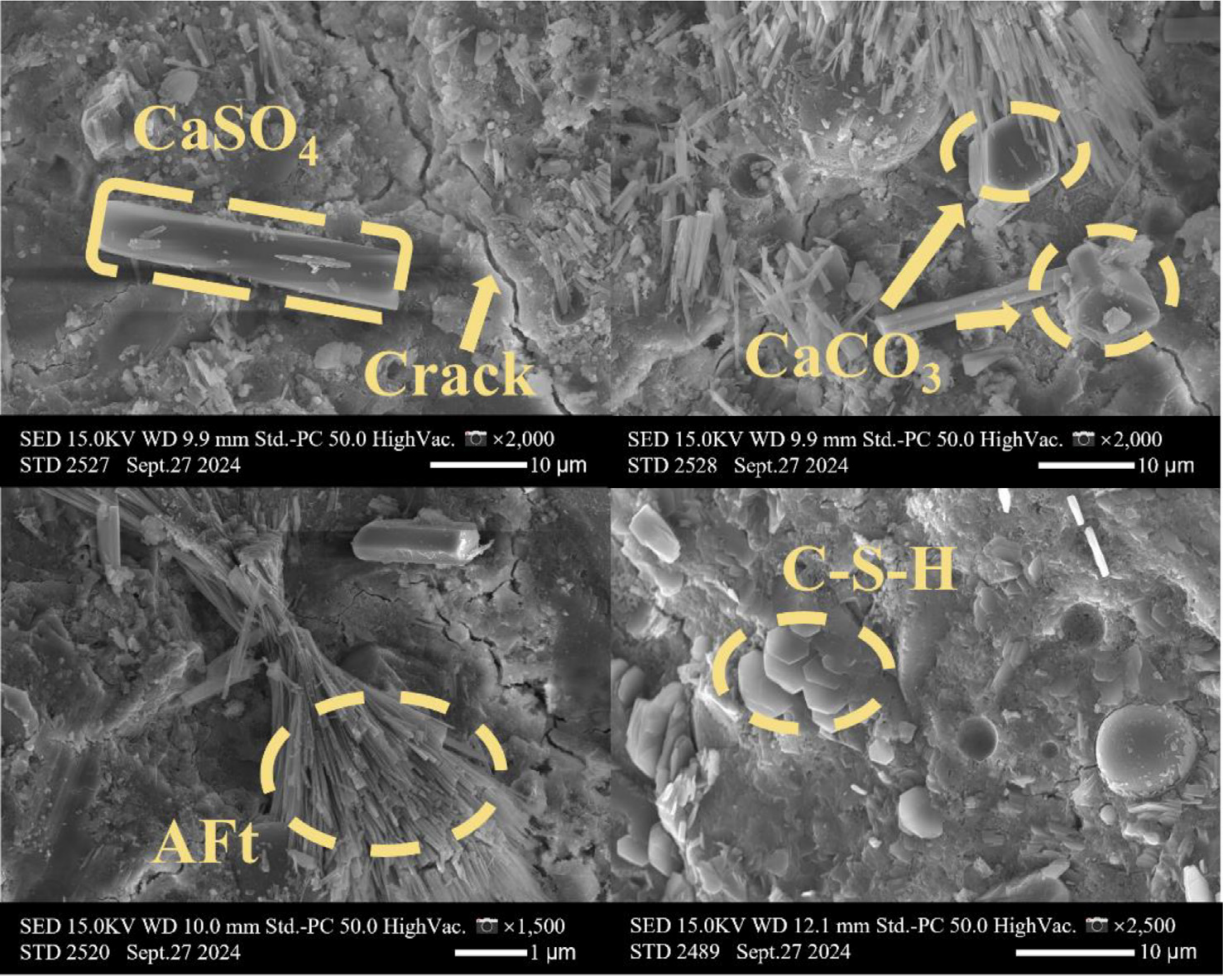
SEM images of hydration products of cement under the influence of sulfate and bicarbonate ions (Group 8) at 28 days.

**Fig. 18:**
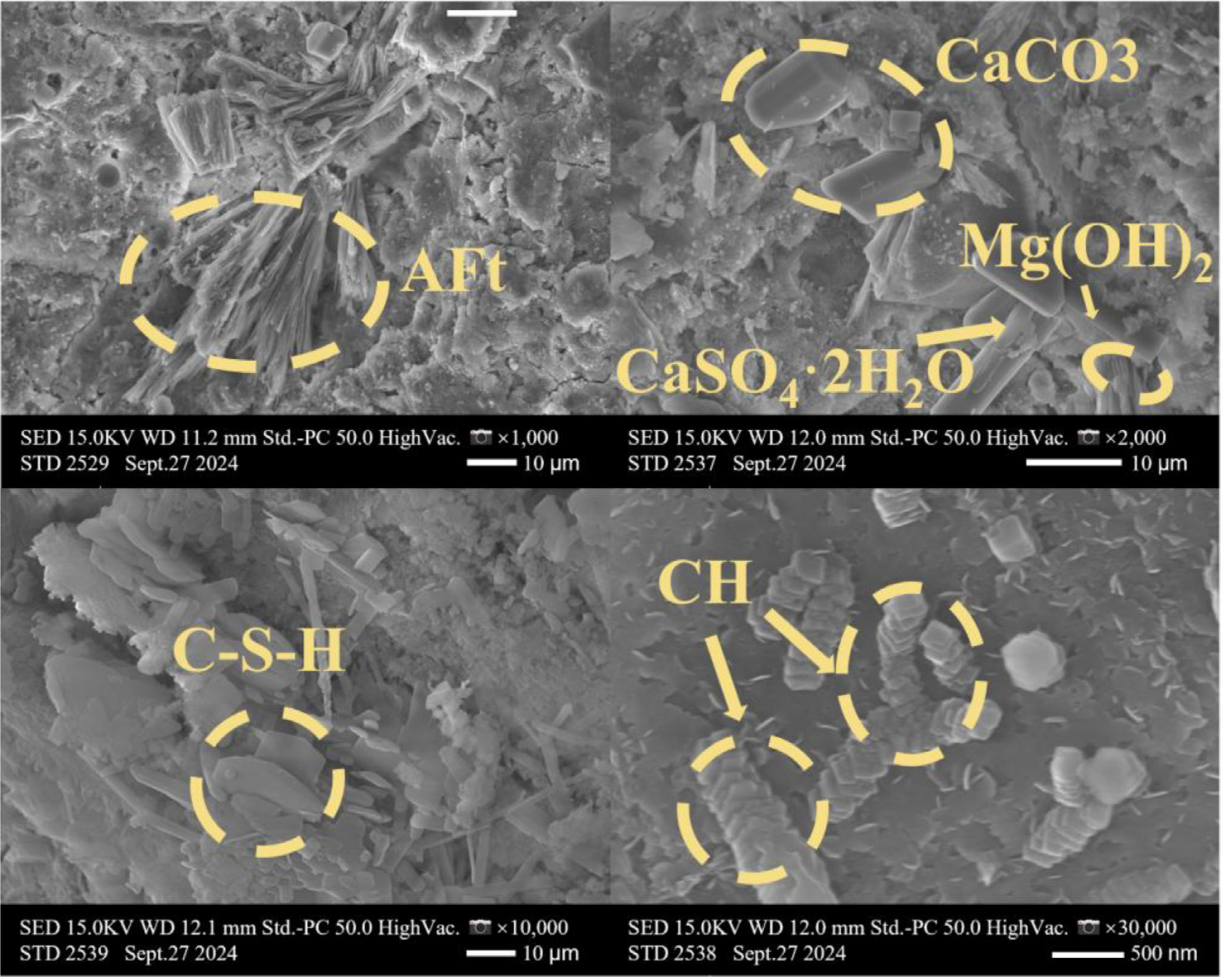
SEM images of hydration products of cement under the influence of sulfate, magnesium, and bicarbonate ions (Group 9) at 28 days.

**Fig. 19:**
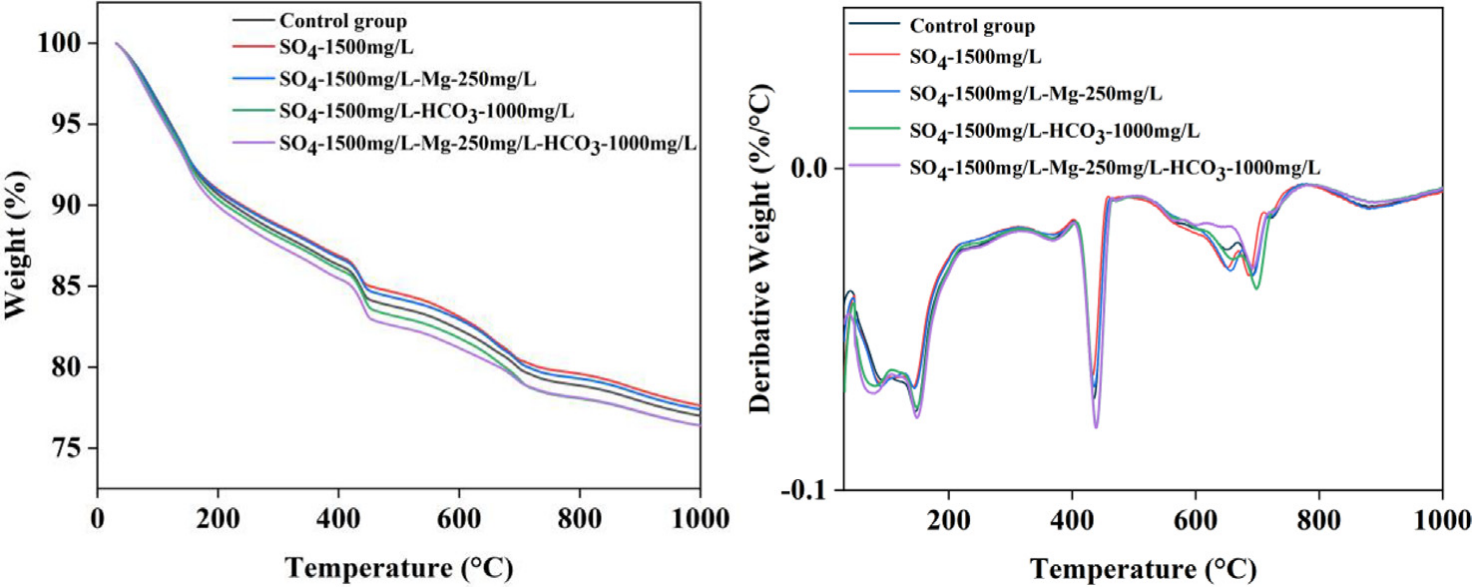
DTG-DTG curves showing the effects of various ions on cement at 28 days.

**Fig. 20:**
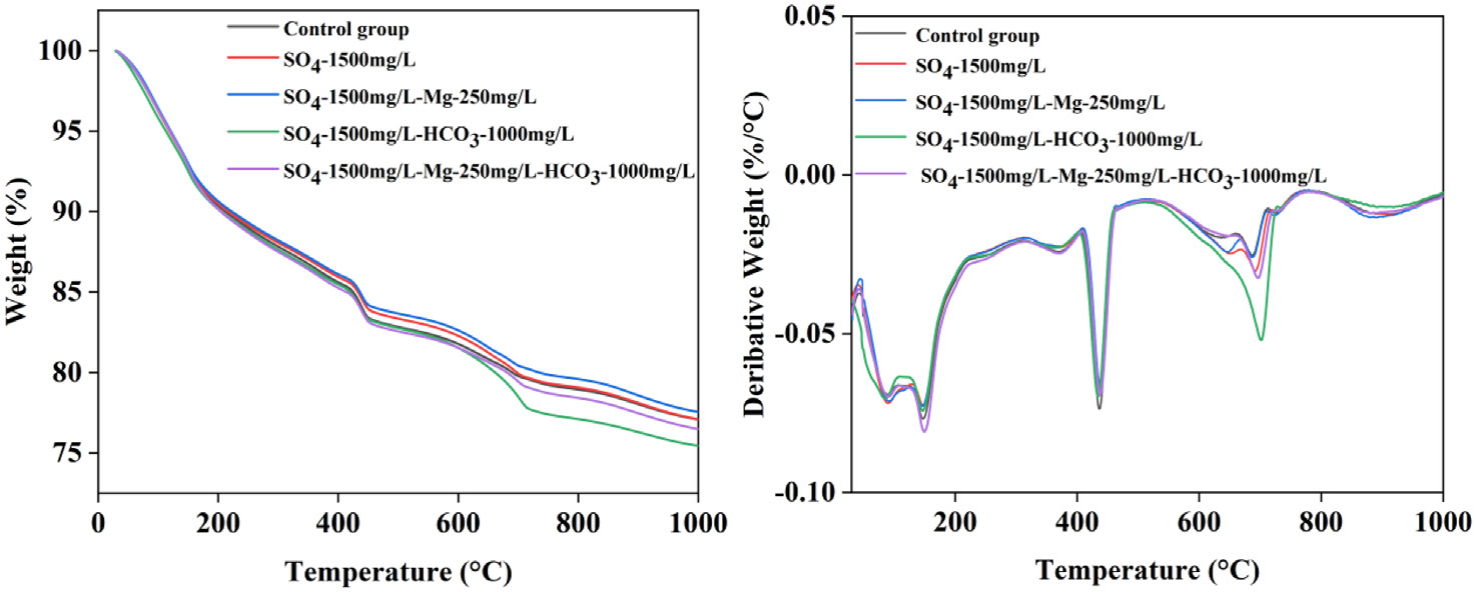
DTG-DTG curves showing the effects of various ions on cement at 90 days.

## 3 Experimental Design, Materials and Methods

### 3.1 Experimental design

The sulfate ion solution, magnesium ion solution, and bicarbonate ion solution used in the experiments were prepared using anhydrous sodium sulfate, anhydrous magnesium sulfate, and sodium bicarbonate, respectively. The water-to-cement ratio for the experiments was set at 0.4, and cement cube samples measuring 50 mm × 50 mm × 50 mm were produced.

### 3.2 Raw materials

The cement clinker used in this study was Type I Portland cement, classified as 42.5. The elemental composition of the cement clinker was analyzed using X-ray fluorescence (XRF) testing, and the results are presented in Table 3.

**Table 3:** Oxide composition of Cement, FA and GBFS (%).

Oxide	SiO_2_	Al_2_O_3_	Fe_2_O_3_	CaO	MgO	SO_3_	Other minor oxides
Cement	14.42	4.77	3.10	71.58	1.72	2.64	1.77

### 3.3 Methods

#### 3.3.1 Method for testing hydration heat

The hydration heat of the cement paste was measured using a TAM-Air isothermal calorimeter produced by TA Instruments. Prior to the experiment, the calorimeter was turned on 8 hours in advance to allow the internal temperature to stabilize at room temperature (25°C). Following the standard procedure (GB/T 1346-2011) [2], a solution or distilled water was added to prepare the cement paste in a cement mixer. A 10 g sample of the paste (with an error margin of ±0.05 g) was then weighed using an ampoule and placed into the calorimeter alongside a reference water sample to measure the hydration heat release of the paste.

#### 3.3.2 Method for testing compressive strength

The cement samples were prepared according to the requirements of GB/T 17671-2021 [3], with a water-to-cement ratio of 0.4. After mixing in a planetary mixer, the samples were molded on a vibrating table to form standard cube specimens (50 × 50 × 50 mm). The specimens were then cured in a standard curing chamber at a temperature of 20 ± 1°C and a humidity of not less than 90% until the specified curing age. After curing, the samples were removed for testing, and the compressive strength was measured using a YAW-300 electro-hydraulic servo pressure testing machine.

#### 3.3.3 X-ray diffraction test

X-ray diffraction (XRD) analysis was conducted using a SmartLab SE X-ray diffractometer produced by Rigaku Corporation in Japan, aimed at investigating the phase composition and crystal structure of the samples. The scanning angle was set within the range of 5° to 80°, with a scanning rate maintained at 5°/min.

#### 3.3.4 Scanning electron microscope test

The experiments employed a Hitachi S4800 scanning electron microscope (SEM) produced by Hitachi, Japan. The samples were directly mounted on conductive adhesive and coated with a thin layer of gold, without any backscattering treatment. The analysis focused on the morphology of the samples. This equipment features a maximum resolution of 1.4 nanometers, with magnification adjustable from 20 × to 800,000 ×, and an acceleration voltage range of 0.5 to 30 kilovolts.

#### 3.3.5 Method for thermogravimetric analysis

The solid-phase powder obtained after ethanol extraction was subjected to thermogravimetric (TG/DTG) analysis using a STA449C thermal analyzer produced by Netzsch, Germany. The sample mass was approximately 20 mg, and the tests were conducted under an argon atmosphere, with a temperature range of 35°C to 1050°C and a heating rate of 10°C/min.

## Data Availability

Experimental Dataset of the Ions in Mine Water on Mechanical Strength and Microstructure of Portland Cement (Original data).
